# Reliability of Health Information on the Internet: An Examination of Experts' Ratings

**DOI:** 10.2196/jmir.4.1.e2

**Published:** 2002-01-17

**Authors:** Mark Craigie, Brian Loader, Roger Burrows, Steven Muncer

**Affiliations:** ^1^Department of Applied PsychologyUniversity of DurhamStockton CampusUK; ^2^Centre for Informatics Research and ApplicationsUniversity of TeessideUK; ^3^Centre for Housing PolicyUniversity of YorkUK

**Keywords:** Newsgroup, Internet, rating information, reliability, reproducibility of results, statistics, quality control

## Abstract

**Background:**

The use of medical experts in rating the content of health-related sites on the Internet has flourished in recent years. In this research, it has been common practice to use a single medical expert to rate the content of the Web sites. In many cases, the expert has rated the Internet health information as poor, and even potentially dangerous. However, one problem with this approach is that there is no guarantee that other medical experts will rate the sites in a similar manner.

**Objectives:**

The aim was to assess the reliability of medical experts' judgments of threads in an Internet newsgroup related to a common disease. A secondary aim was to show the limitations of commonly-used statistics for measuring reliability (eg, kappa).

**Method:**

The participants in this study were 5 medical doctors, who worked in a specialist unit dedicated to the treatment of the disease. They each rated the information contained in newsgroup threads using a 6-point scale designed by the experts themselves. Their ratings were analyzed for reliability using a number of statistics: Cohen's kappa, gamma, Kendall's W, and Cronbach's alpha.

**Results:**

Reliability was absent for ratings of questions, and low for ratings of responses. The various measures of reliability used gave conflicting results. No measure produced high reliability.

**Conclusions:**

The medical experts showed a low agreement when rating the postings from the newsgroup. Hence, it is important to test inter-rater reliability in research assessing the accuracy and quality of health-related information on the Internet. A discussion of the different measures of agreement that could be used reveals that the choice of statistic can be problematic. It is therefore important to consider the assumptions underlying a measure of reliability before using it. Often, more than one measure will be needed for "triangulation" purposes.

## Introduction

The importance of the Internet for contemporary public health has been acknowledged for some time. People have used the Internet for many years to access health-related information. Pallen points out that, although health professionals originally assumed that health-related Internet sites would be something used by themselves for research, consultation with colleagues, continuing education, and library work, this concept has been extended and modified [1]. Now the importance of the Internet as a source for health information for the layperson is increasingly acknowledged [2,3].

The Graphics, Visualization & Usability Center at Georgia Institute of Technology estimated that 27% of female Internet users and 15% of male Internet users use the Internet to get medical information on a regular basis [4]. These figures have now mushroomed to 63% of women on-line and 46% of men on-line [5]. The growth rate in lay use of Internet health sites is rapid: a Harris Interactive study estimated that, from April 1999 to September 1999, the number of Internet users in America accessing health information increased from 60 million to 70 million [6]. Given this large-and-growing audience, the quality of medical information on the Internet has become an increasingly-important concern, as expressed in Eysenbach and Diepgen and the associated commentaries [7]. This is particularly true given that approximately half of the Internet users surveyed in the Fox et al [5] study said that they had acted upon information gleaned from the Internet to change their health behavior, including, if they were ill, changing aspects of their treatment and care. Such information may be a matter of life and death [8]. There have been warnings that a lot of the information on the Internet is either harmful or misleading [9]. Studies that have evaluated the information on the Internet have often found it to be incomplete and sometimes dangerous [2,7,10,11]. The concerns of lay users of the Internet reflect the concerns of medical professionals: 86% of Internet users are concerned about the reliability of the health information available on the Internet [5]. Despite these concerns, however, 52% of people who regularly use health sites on the Internet consider the information on those sites to be credible, particularly people with low levels of formal education [5]. In addition, most Internet users gain access to health sites by Internet search rather than recommendation by a professional [5]. It is therefore important to have a solid empirical basis for selecting the criteria for rating medical sites on the Internet, whether it is lay users or medical professionals doing the rating.

Leaving aside the question of whether a reliance on medical opinion will "dismiss the input of non medical readers" [12], we would argue that a greater problem is that some of the studies using medical raters suffer from an overreliance on one medical opinion. For example, no statistics are given about the agreement between medical raters and Sandvik [11] explicitly acknowledges this weakness of his study: "A stronger design would be to include judgements from several experts to allow assessment of judge's reliability." The present study attempts to overcome this weakness by asking more than one medical expert to categorize the information given on a well-used newsgroup dealing with a chronic illness. The illness has a relatively-high prevalence and is one seen regularly in both primary care and more-specialized medical services. It is an illness for which misleading information would be harmful and potentially fatal. The categories used were designed by our experts and reflected the current importance of evidence-based medicine.

## Methods

### Participants

The 5 medical experts who participated were all doctors experienced in the treatment of the chronic illness chosen. They worked together in the same specialist unit and all had at least 5 years experience in treatment of the chosen illness.

### Materials

The material to be categorized came from a newsgroup used mainly by nonprofessional medical sufferers of the illness. Overall, there were 61 threads (series of connected messages), selected from a week's posting because they contained medically-related information, to be examined by at least one medical expert; however a random sample of 18 threads was assessed by all 5 experts. These 18 threads form the basis of this report.

Each thread consisted of a start message; usually in the form of a question; and a number of responses. Both the start message and the responses were rated using a coding scheme devised by the medical experts. For start messages, there was a 6-part scheme: A = excellent; B = less good but with some details; C = poor with little detail; D = vague; E = misleading or irrelevant; F = incomprehensible. The responses were also coded according to a 6-part scheme: A = evidence based, excellent; B = accepted wisdom; C = personal opinion; D = misleading, irrelevant; E = false; F = possibly dangerous.

### Statistical Analysis

There are 3 main ways (kappa, gamma, and Kendall's W) to analyze the agreement of judges rating the threads from the Internet. Perhaps the most familiar to medical researchers and practitioners is Cohen's kappa. We present the version of kappa described in Siegel & Castellan [13] in which a single kappa statistic reflects the agreements across all 5 judges; this statistic is equivalent to the average of all kappa statistics calculated pair wise. However, this statistic assumes the data is nominal in measurement. The data we have is ordinal (ie, the scale from A to F has a fixed order) and so Cohen's kappa, although familiar and often used, is inappropriate for this data. We include it only because it is so often used for this type of data in other studies.

There is a choice of the most appropriate statistic to analyze such data. One could use a weighted-kappa procedure, but this statistic is controversial because the values of the weights for each level are arbitrary [14]. The gamma statistic [13] is related to the weighted kappa statistic and so is presented instead for comparison with the unweighted kappa values. This statistic has been computed for all pair-wise combinations of experts, and the Bonferroni adjustment for multiple comparisons has been applied to the significance levels. Perhaps a more-powerful statistic is Kendall's W, which is similar to the unweighted kappa value in that one statistic represents the overall agreement between the 5 experts. Kendall's W is linearly related to the average rank correlations between ratings assigned by the judges to the threads [13], so it ranges from 0 to 1; hence, it is relatively easy to interpret and can be converted to a **c** ^2^ statistic to test for significance. It also provides us with a relatively-powerful measure of average agreement among our experts, unlike the average of pair-wise rank correlations.

## Results

### Start Messages

For the start messages, the kappa statistic was 0.024; this value was not significant ( *z*= 0.45, *P*> .05). It is generally accepted in medical circles that a kappa of over 0.75 represents excellent agreements and between 0.4 and 0.74 represents fair-to-good reliability [15]. However, distribution and base rate can affect the kappa statistic [16]. In this case, there is poor agreement between the experts using kappa as a measurement of agreement. However, some power is lost treating ordinal data as nominal, although a similar result occurs if the gamma statistic is used. Only 1 of the 10 pair-wise gamma statistics was significant, and this was negative (Table 1), showing significant *dis* agreement between those 2 experts (gamma = -0.659, *P*< .01)! The other gamma statistics were all positive and ranged from 0 to 0.475. There is no agreement between raters using this measure. The value of Kendall's W for the ratings of start messages, however, tells a different story. It reflects a modest, but highly-significant, amount of agreement between judges (W = 0.266, **c** ^2^(4) = 19.2, *P*< .001). We suspect that this statistic is due mainly to the single strongly-negative relationship between the ratings of 2 experts. If the agreements of the other experts were weak and randomly distributed, then a single value would dominate the W statistic and so produce a significant result. As W cannot be negative (more than 2 judges cannot all disagree with each other), the result will be a statistic that is misleading. It is therefore important that researchers consider both overall and pair-wise statistics when assessing inter-rater reliability.

**Table 1:** Gamma Statistics for the Rating of Start Messages

**Expert 2**		**Expert 1**				
		**1**	**2**	**3**	**4**	**5**
	**1**	1	0.000	0.181	0.247	-0.659**
	**2**	0.000	1	0.345	0.262	0.368
	**3**	0.181	0.345	1	.475	0.250
	**4**	0.247	0.262	0.475	1	0.409
	**5**	-0.659**	0.368	0.250	0.409	1
		*P < .05	**P < .01	***P < .001		

### Replies

Overall, the results for the agreement of rating of responses to these start messages were somewhat better. The kappa statistic for these ratings was 0.243 and was significant ( *z*= 5.49, *P*< .001). Individual agreement between raters, as assessed by the gamma statistic, ranged from a low of 0.311 to a high of 0.730 (Table 2). The majority of gamma values were significant; however, 3 failed to reach significance (maximum nonsignificant value was 0.431). There is general agreement, but it is not as high as one might hope. The W statistic, however, was extremely low and only just significant (W = 0.037, **c** ^2^(4) = 10.4, *P*< .05). The overall pattern of agreement is not clear, even though individual pairs of experts appear to agree with each other. This strongly suggests that there are a number of different pairings within our expert panel that contradict each other.

**Table 2:** Gamma Statistics for the Rating of Replies

**Expert 2**		**Expert 1**				
		**1**	**2**	**3**	**4**	**5**
	**1**	1	0.431	0.377	.730***	0.602*
	**2**	0.431	1	0.578***	.621***	0.311
	**3**	0.377	0.578***	1	.592***	0.504**
	**4**	0.730***	0.621***	0.592***	1	0.690***
	**5**	0.602*	0.311	0.504**	.690***	1
		*P < .05	**P < .01	***P < .001		

A more-imaginative approach to the problem of assessing reliability and validity for ratings of this type was suggested by an anonymous reviewer. The first suggestion was to treat the data as interval level rather than ordered categorical, which would allow greater flexibility in analysis. Furthermore, this approach is relatively common in the social sciences and more particularly in psychometric research. The second suggestion was that a simple and effective way of presenting the data would be to give the Spearman rank order correlation for raters. We present these for the ratings of the replies in Table 3. The third suggestion was that we treat the data like psychometric test data and take each rating as similar to an item on a test instrument. We can then calculate Cronbach's alpha and use this as a measure of reliability. Further we can then use the Spearman-Brown prophecy formula to predict how the reliability of the ratings would increase if we had different numbers of raters. This formula is used in psychometric research to estimate the increases in reliability expected if the number of items is increased.

**Table 3:** Spearman Rank Order Correlations for Replies

**Expert 2**		**Expert 1**				
		**1**	**2**	**3**	**4**	**5**
	**1**	1	0.296*	0.248*	0.519***	0.416***
	**2**	0.296*	1	0.454***	0.538***	0.233*
	**3**	0.248*	0.454***	1	0.452***	0.334**
	**4**	0.519***	0.538***	0.452***	1	0.516***
	**5**	0.416***	0.233*	0.334**	0.516***	1
		*P < .05	**P < .01	***P < .001		

In this case, the Cronbach's alpha for the 5 doctor's ratings of the replies was 0.78. This reliability, however, would be increased to 0.876 by doubling the number of raters to 10 and to 0.914 if we increase the number of raters to 15. If we only have 2 raters, the reliability is reduced to a very-worrying 0.59.

### Increasing Reliability

For medical evidence of this type, we would want to have information that is as reliable as possible; 5 doctors as in our example may be too few. The reliability can be increased by increasing the number of items to be rated as well as by increasing the number of raters. The Spearman-Brown formula is limited to estimating differences in one dimension - in this case, the number of raters. Brown [17] has suggested the use of generalizability theory that can provide answers in more than one dimension; that is, what would happen to reliability if we increase the number of raters and the number of items rated?

## Discussion

Overall, the results suggest that there is a fair degree of disagreement between medical experts when they are asked to rate medically-related postings from the Internet. In this case, the experts were using a system that was devised by them, so any possibility of this result being forced on them by a poor or deliberately-misleading category system is negated. We acknowledge that the start-message coding is less important as it deals with questions rather than answers, includes a small sample, and its coding seems by its nature to be less precise, which may explain the very-low levels of agreement. The rating of responses, however, seems to us to use sensible and relatively-transparent categories. The agreement between response ratings is still relatively poor, and certainly not consistent across all the experts.

One particularly interesting finding was the divergence of the different statistics used to measure agreement in the same ratings. It seems that the choice of a statistic to measure the agreement of judges in this sort of research could be problematic. Consideration of the power of a statistic and the use of pair-wise versus overall statistics are the two main issues. In particular, we have shown that it is possible to achieve a reasonably-high level of agreement with an overall test when individual pair-wise statistics show no agreement or significant disagreement (as was the case for start messages). We have also shown that overall statistics can conflict with pair-wise statistics when there are subgroups within the raters who agree with each other, but disagree with the other subgroups. This was the case with the replies: the overall level of agreement was very low, but individual pair-wise statistics showed high agreement between pairs of raters. The selection of a homogeneous group of experts (such as ours) did not seem to eliminate this problem.

The anonymous reviewer's suggestion for adopting psychometric techniques to look at the reliability of the raters is interesting, and we believe could be a valuable procedure for the future. Both factor analysis and latent structure analysis [18] could also be usefully employed with this sort of data but would require larger samples than we have here.

These results call into question the numerous studies that have claimed to show that the information on the Internet is of poor quality, and suggest that future studies should employ more than one rater. That one expert fails to agree with the Internet is perhaps less important than that several experts disagree with each other. It is possible that training or other resources might increase agreement between experts, and future research could consider this. Any measure producing a greater agreement between raters of Internet sites could have great benefits to medical and nonmedical users of the Internet alike.

## References

[1] Pallen M (1995). Introducing the Internet. BMJ.

[2] Impicciatore P, Pandolfini C, Casella N, Bonati M (1997). Reliability of health information for the public on the World Wide Web: systematic survey of advice on managing fever in children at home. BMJ.

[3] Widman L E, Tong D A (1997). Requests for medical advice from patients and families to health care providers who publish on the World Wide Web. Arch Intern Med.

[4] Graphics, Visualization & Usability Center at Georgia Institute of Technology (1998). GVU's 10th WWW user survey.

[5] Fox S, Rainie L, Horrigan J, Lenhart A, Spooner T, Burke M The online health care revolution: How the web helps Americans take better care of themselves.

[6] Taylor H Explosive growth of "cyberchondriacs" continues.

[7] Eysenbach G, Diepgen T L (1998). Towards quality management of medical information on the internet: evaluation, labelling, and filtering of information. BMJ.

[8] Weisbord S D, Soule J B, Kimmel P L (1997). Poison on line--acute renal failure caused by oil of wormwood purchased through the Internet. N Engl J Med.

[9] Gustafson D H, Robinson T N, Ansley D, Adler L, Brennan P F (1999). Consumers and evaluation of interactive health communication applications. The Science Panel on Interactive Communication and Health. Am J Prev Med.

[10] Eysenbach G, Sa E R, Diepgen T L (1999). Cybermedicine. Interview by Clare Thompson. BMJ.

[11] Sandvik H (1999). Health information and interaction on the internet: a survey of female urinary incontinence. BMJ.

[12] Whatling P (1999). Having non-medical readers of papers on internet will enhance peer review. BMJ.

[13] Siegel Sidney, Castellan N John (1988). Nonparametric Statistics for the Behavioral Sciences.

[14] Maclure M, Willett W C (1987). Misinterpretation and misuse of the kappa statistic. Am J Epidemiol.

[15] Landis J R, Koch G G (1977). The measurement of observer agreement for categorical data. Biometrics.

[16] Byrt T, Bishop J, Carlin J B (1993). Bias, prevalence and kappa. J Clin Epidemiol.

[17] Brown JD (1999). (1999). Relative importance of persons, items, subtests and languages to TOEFL test variance. Language Testing.

[18] Uebersax, JS (1993). (1993). Statistical modeling of expert ratings on medical treatment appropriateness. American Statistical Association 1993;88:421-427. Journal of the American Statistical Association.

